# The Injection of Ghrelin (OXE-103) Improves Subacute Concussion Symptom Burden and Quality of Life

**DOI:** 10.1089/neur.2025.0038

**Published:** 2025-05-16

**Authors:** Michael Rippee, Michael Wyand, Jamie Chen, Amelia Kirchhoff-Rowald, Suzanne Hunt, Vishal Bansal

**Affiliations:** ^1^ University of Kansas Medical Center, Kansas City, Kansas, USA.; ^2^ Oxeia Biopharmaceuticals, Boston, Massachusetts, USA.

**Keywords:** concussion, drug therapy, ghrelin, mild traumatic brain injury

## Abstract

Concussions remain the leading form of traumatic brain injury. Despite this, there is a paucity of pharmacologic and evidence-based treatments. The objective of this study was to investigate the benefit of Ghrelin (OXE-103) as a novel treatment for subacute concussion. The study consisted of an open-label treatment arm (OXE-103) with a nontreatment concurrent control arm receiving standard of care (SOC-only). Participants had a documented concussion, within 28 days of injury, and a Post-Concussion Symptom Scale (PCSS) of 20 or more. A total of 19 subjects completed the study: 13 treatments and 6 SOC. Treatment consisted of OXE-103 40 μg/kg twice daily by self-injection for 14 days. Main Outcome Measures were change in PCSS and Quality of Life after Brain Injury–Overall Scale (QOLIBRI-OS). Outcome measures were assessed at days 1, 4, 8, 11, 15, 21, and 44. A secondary outcome was 20% improvement on either which was considered a clinically meaningful response. We found a decrease in PCSS from baseline with OXE-103 (median −34 [interquartile range {IQR}: −44, −24]) at day 44 versus SOC (median −7 [IQR: −22, 16]) at day 44. We also found an improvement in QOLIBRI-OS from baseline with OXE-103 (median 21 [IQR: 12.5, 50]) at day 44 versus SOC (2 [IQR: –25, 20.8]) at day 44. 85% (95% confidence interval [CI]: 53, 98) of subjects treated with OXE-103 had a clinically meaningful response at day 44 on PCSS versus 33% (95% CI: 4, 78) of subjects in the SOC arm. When looking at improvement in QOLIBRI-OS, 85% (95% CI: 53, 98) of subjects treated with OXE-103 had a clinically meaningful response at day 44 versus 33% (95% CI: 4, 78) in the SOC arm. We conclude that subjects treated with OXE-103 showed improved PCSS and QOLIBRI-OS scores compared to those receiving only standard therapy. We recognize the limitations of this study, including small sample size and lack of randomization. The results indicate that OXE-103 is a potential therapeutic agent to treat patients with ongoing concussion symptoms. A larger, multicenter, randomized, placebo-controlled trial would be an important next step.

## Introduction 

Concussions remain the leading form of traumatic brain injury (TBI) with an estimated incidence of at least six to seven million concussions annually, including both reported and unreported injuries.^1,2^ Despite this, there is a paucity of pharmacologic and evidence-based treatments. Currently, standard of care (SOC) includes a combination of cognitive rest, physical therapy and a gradual return to normal activity. As is common with other neurological conditions, both diagnosis and response to treatment strategies are exclusively linked to symptom assessment and symptom management. This reflects the current absence of biomarkers or imaging modalities to diagnose and monitor concussion injury.

OXE-103 is a 28 amino-acid polypeptide hormone produced semisynthetically and is identical to endogenous acylated ghrelin secreted by the stomach. Ghrelin was long thought to be exclusively an orexigenic hormone, however, the physiological and pharmacological effects of ghrelin are diverse, pleiotropic, and include neuroprotective properties. Ghrelin receptors (GHSR-1) are present throughout the brain, where its binding increases mitochondrial protein uncoupling protein 2 (UCP-2) expression and “uncouples” oxidative phosphorylation. This leads to an increase in mitochondrial generation and attenuates toxic reactive oxygen species (ROS) formation.^3,4^ Furthermore, ghrelin directly increases the synaptic density of the hippocampus and is associated with improved memory and neurocognition.^3,5^ A recent observational human study identified a positive correlation between endogenous serum ghrelin levels and improved neurocognitive function following mild TBI.^3^ OXE-103 has been demonstrated to be well tolerated via subcutaneous administration with a 300-patient safety database from previous studies in non-neurological indications.^6^

Concussions cause a well-established neurometabolic cascade resulting in a massive neuronal efflux of potassium and influx of calcium.^7^ Concomitantly, sudden neuronal depolarization results in a spike of glutamate, which can be toxic to surrounding neuronal tissue. This cascade of electrolyte abnormalities leads to a significant increase in cellular active transport, resulting in expenditure of energy, cellular glucose deprivation, and an overall energy crisis. Mitochondrial respiration is disrupted, and formation of adenosine triphosphate (ATP) is impaired, collectively leading to pathological oxidative stress, inflammation, and the generation of ROS. In addition, concussions may also cause a disruption of axonal transport, axonal swelling, and a decrease in neuronal connectivity.^7^ These pathological changes can persist past the acute phase due to ongoing oxidative damage and altered neurotransmission.^7^ Collectively, these neurometabolic changes cause a composite of post-concussive symptomatology, quantified using various scoring metrics.

While upward of 80% of concussions resolve within a few weeks, those with persisting symptoms can experience high levels of disability.^8^ Current pharmacologic interventions are limited and normally only target a specific symptom or subset of symptoms, such as headache, disequilibrium, insomnia, or nausea. The underlying mitochondrial and neurometabolic pathophysiology, however, is not abrogated. This population represents a significant unmet medical need. Given the direct effect of ghrelin on the metabolic pathways of the brain and specifically within the hippocampus, we hypothesized that ghrelin therapy could decrease symptoms in patients having suffered a concussion with subacute symptomatology.

## Methods

Subjects with a documented diagnosis of concussion (using 2023 American Congress of Rehabilitation Medicine diagnostic criteria) and 18–60 years of age were recruited within 28 days of their injury. Subjects were recruited from a comprehensive concussion clinic at a tertiary health system. Exclusion criteria included preexisting neurological conditions (including cognitive dysfunction), endocrine abnormalities such as diabetes mellitus, excess or deficiency of growth hormone, cortisol, or prolactin, and pregnancy. This study was conducted under the protocols and in accord with the ethical standards of the responsible institutional review board (IRB).

The 22 symptom Post-Concussion Symptom Scale (PCSS) severity across cognitive, emotional, and physical domains has been used extensively to diagnose and monitor acute concussive injury particularly in athletes.^9,10^ A PCSS of 20 or greater has been reported to be associated with the likelihood of patients remaining persistently symptomatic.^11,12^ Thus, a requirement for enrollment was a PCSS score of 20 or greater to enrich the population for persistent symptoms. The PCSS was also used as the primary assessment tool for response to treatment in this study, given its extensive use in clinical settings to monitor symptom improvement. Quality of life was assessed using the Quality of Life after Brain Injury–Overall Scale (QOLIBRI-OS) tool developed specifically for TBI.^13,14^

Subjects who had a 20% or greater improvement in PCSS and/or QOLIBRI-OS responses were considered to have a clinically significant response to treatment and were labeled as “responders.” This was felt by the research team to represent a significant clinical change that a subject could observe. Relative change in PCSS and QOLIBRI-OS was felt to be a better representation of similar changes between subjects with different initial scores on these scales.

QOLIBRI-OS, like other instruments measuring quality of life, is converted to a scale of 0–100 by subtracting 1 from the mean score and then multiplying by 25. This can lead to values of 0, which does not allow for calculating responder status, as 0 cannot be used as a denominator. When calculating responder status for QOLIBRI-OS, we wanted to be consistent in using an improvement of 20% or greater to indicate a clinically significant response. Therefore, we used the sum of responses for the six QOLIBRI-OS questions in our responder calculations. Several participants had QOLIBRI-OS values at the beginning of the trial, which precluded us from calculating the percent change in those circumstances.

The study was originally designed as a randomized placebo-controlled trial. Enrolled subjects would be given OXE-103 40 μg/kg or placebo by subcutaneous self-injection twice daily for 14 days. Enrollment goals were 20 subjects per arm. After IRB approval, recruitment opened and only two subjects were enrolled in the first year, only one of which was randomized. Due to slow enrollment, the decision was made to change the design to an open-label trial. Participants received either SOC-only or SOC and OXE-103 injections. SOC treatment of both groups included vestibular physical therapy as well as speech and cognitive therapy. Patients with a PCSS score of 20 or greater at the time of presentation were approached for screening and enrollment in the OXE-103 arm. If they declined to receive OXE-103, enrollment in the SOC-only arm was discussed.

Starting with day 1, the treatment cohort received OXE-103 40 μg/kg by subcutaneous self-injection twice daily for 14 days. The study drug was maintained and dispensed by the institution’s Investigational Pharmacy. Subjects received an eight-day supply of syringes preloaded with OXE-103. The treatment cohort received the second set of syringes with OXE-103 at the day 8 visit.

At each study visit (days 1, 8, 15, 21, and 44), subjects were asked to complete the PCSS and QOLIBRI-OS questionnaires. Subjects also completed the PCSS and QOLIBRI-OS online on days 4 and 11.

The institutional review board at the University of Kansas Medical Center provided approval and oversight of the study. Study data were collected and managed using REDCap (Research Electronic Data Capture) tools (ULITR002366). REDCap is a secure, web-based software platform designed to support data capture for research studies, providing (1) an intuitive interface for validated data capture; (2) audit trails for tracking data manipulation and export procedures; (3) automated export procedures for seamless data downloads to common statistical packages; and (4) procedures for data integration and interoperability with external sources.^15,16^ BioPortal ontologies were used when documenting medical history, concomitant medications, and adverse events.^17,18^

Descriptive statistics were used to describe the sample and the observed outcomes. Median and range or interquartile range (IQR) were provided for numeric measures, while counts and percentages were given for categorical data. Plots provide a visual representation of outcomes over time.

## Results

**FIG. 1.**
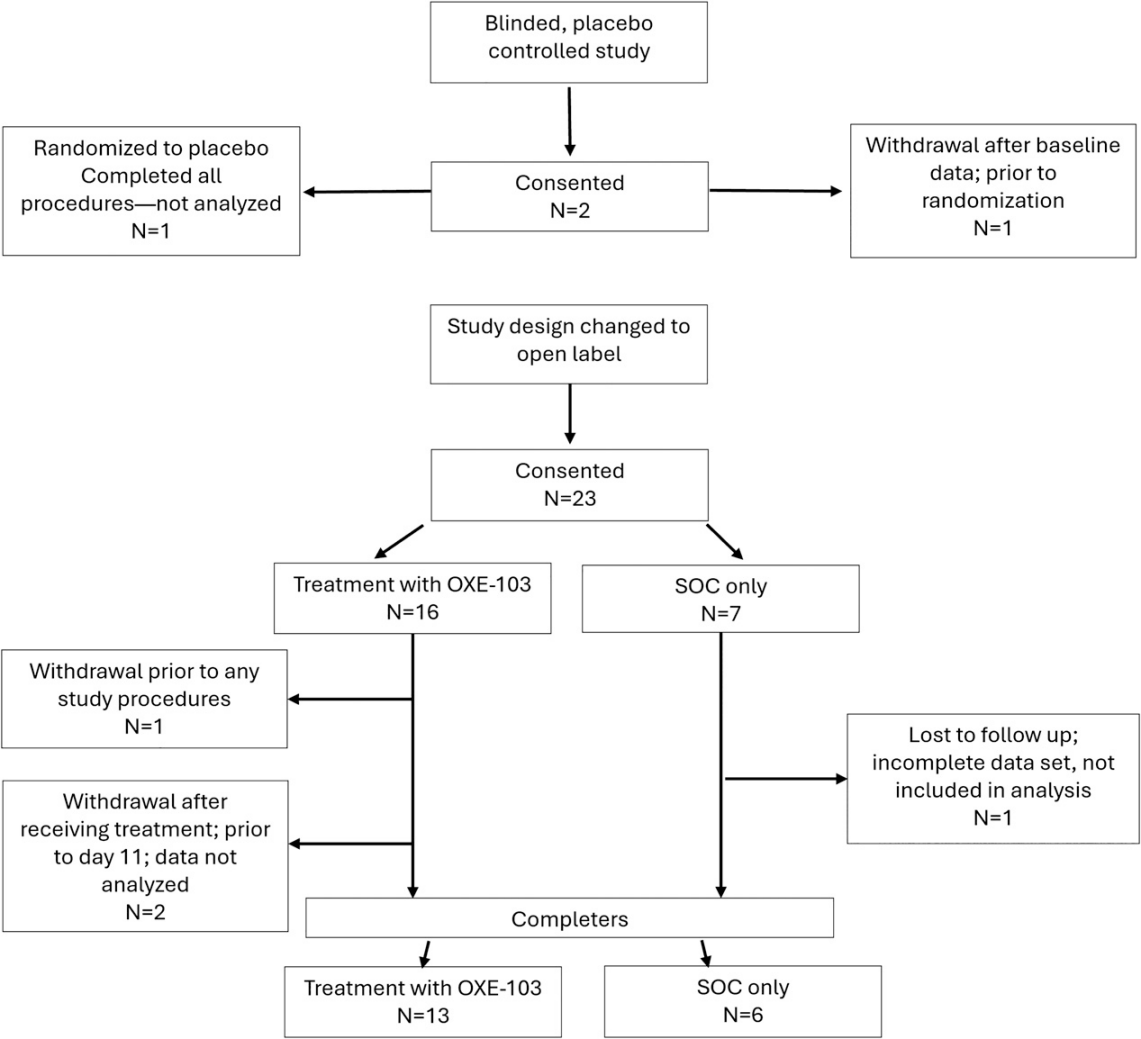
Recruitment/retention diagram.

A total of 25 subjects enrolled with 19 completing all study procedures; 13 received OXE-103 treatment and 6 SOC-only (Table 1). One subject withdrew after consenting but prior to completing any study activities. One subject withdrew after completing baseline activities but prior to receiving any study treatment. Two subjects consented and received some treatment but withdrew before study completion. One SOC-only participant was lost to follow-up. One was randomized to placebo early on and completed the study but is not included in the analysis (Fig. 1).

**Table 1.** Participant Characteristics

Demographic Data	OXE-103 *n* = 13	SOC *n* = 6
Age, median (range)	42 (33–59)	33 (40–49)
Sex, %		
Female	69%	83%
Male	31%	16%
Race, %		
Other	8%	0%
White	92%	100%
Days post injury, median (range)	26 (8–31)	25 (9–30)
Concussion Hx, %	46%	33%
PCSS baseline, median (range)	71 (37–102)	44 (28–93)
QOLIBRI baseline, median (range)	8 (6–21)	13 (6–18)
Mechanism of injury, %		
Fall	53%	17%
Struck	23%	33%
Motor vehicle	15%	50%
Other	8%	0%
Loss of consciousness, %	15%	0%

PCSS, Post-Concussion Symptom Scale; QOLIBRI, Quality of Life after Brain Injury–Overall Scale.

The demographic characteristics of the enrolled are presented in Table 1. Subjects in both groups were between 30 and 60 years of age: OXE-103 treated subjects’ median age was 42 years old (range: 33–59), SOC median age was 33 years old (range: 30–49). The number of days since injury at enrollment was comparable in both groups: OXE-103 median 26 days post injury (range: 8–31), SOC median 25 days post injury (range: 9–30). PCSS scores upon entry into the study were considerably higher than the required score of 20: OXE-103 median 71 (range: 37–102), SOC median 44 (range: 28–93). QOLIBRI-OS was comparable for the two groups at baseline: OXE-103 median 8 (range: 0–63), SOC median 27 (range: 0–50). Subjects recruited were nonathletes with injuries associated with falls, motor vehicle accidents, and being struck. The majority of subjects were female and Caucasian, with approximately half reporting a previous history of concussion.

**FIG. 2.**
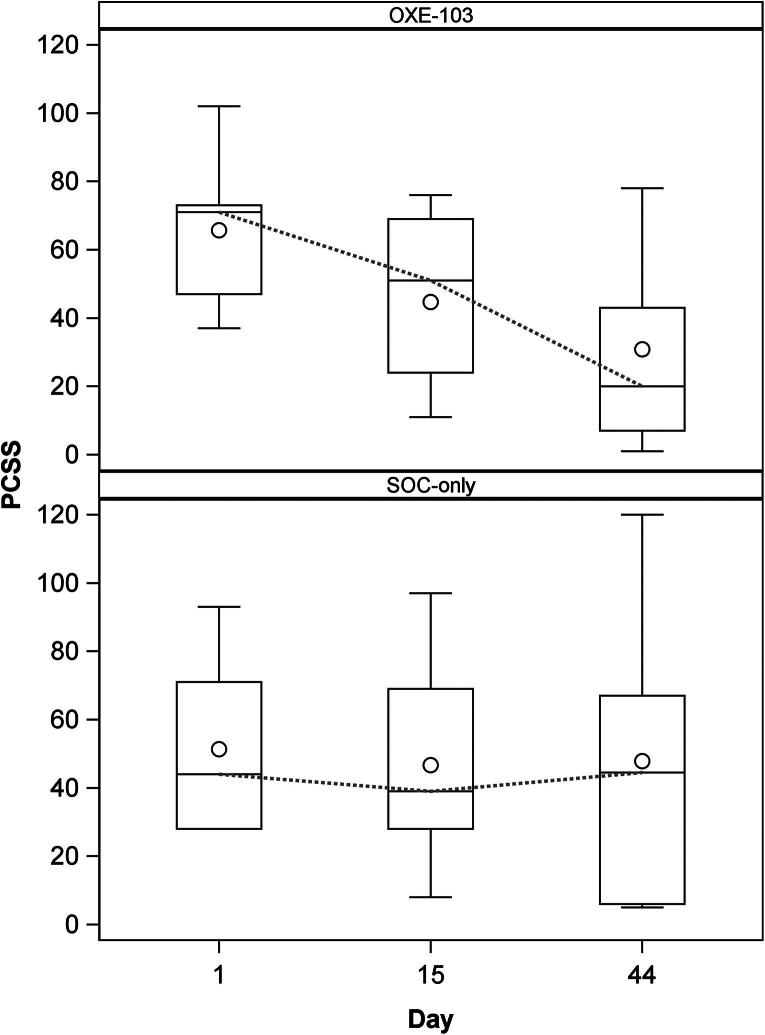
Change in PCSS over time. PCSS, Post-Concussion Symptom Scale.

**FIG. 3.**
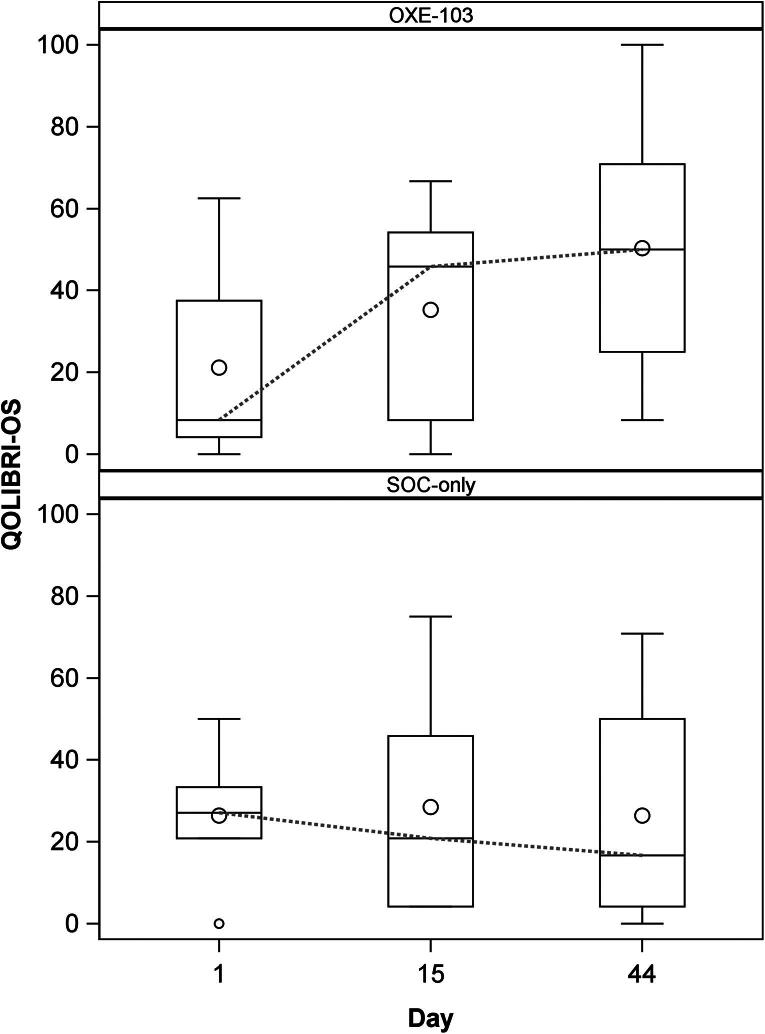
Change in QOLIBRI over time. QOLIBRI, Quality of Life after Brain Injury–Overall Scale.

Due to the small sample size and treatment assigned by participant choice, we elected for a descriptive data analysis. Median and IQR were chosen for reporting observed responses. Negative values for change indicate a decrease in the measure. Median change from baseline in PCSS scores at days 15 and 44, respectively, for the SOC-only group are −1 (IQR: −14, 4) and −7 (–22, 16) versus the OXE-103 group −21 (−30, −6) and −34 (−44, −24) (Table 2 and Fig. 2). Median change from baseline of QOLIBRI-OS scores at days 15 and 44, respectively, for SOC-only are 2 (−17, 25) and 2 (−25, 20.8) versus OXE-103 8 (4.2, 25) and 21 (12.5, 50) (Table 3 and Fig. 3). PCSS and QOLIBRI-OS scores for each individual subject at the specified time points can be found in the Supplementary Data S1.

**Table 2.** Change in PCSS

PCSS	Cohort	
	OXE-103	SOC
*N*	13	6
PCSS, median (IQR)		
Day 1	71 (47, 73)	44 (28, 71)
Day 15	51 (24, 69)	39 (28, 68)
Day 44	20 (7, 43)	45 (6, 67)
Change day 15–day 1	−21 (−30, −6)	−1 (−14, 4)
Change day 44–day 1	−34 (−44, −24)	−7 (−22, 16)

IQR, interquartile range; PCSS, Post-Concussion Symptom Scale; SOC, standard of care.

**Table 3.** Change in QOLIBRI-OS

QOLIBRI-OS	Cohort	
	OXE-103	SOC
*N*	13	6
QOLIBRI-OS, median (IQR)		
** **Day 1	8 (4.2, 37.5)	27 (20.8, 33.3)
** **Day 15	46 (8.3, 54.2)	21 (4.2, 45.8)
** **Day 44	50 (25, 70.8)	17 (4.2, 50)
Change day 15–day 1	8 (4.2, 25)	2 (−17, 25)
Change day 44–day 1	21 (12.5, 50)	2 (−25, 20.8)

IQR, interquartile range; QOLIBRI-OS, Quality of Life after Brain Injury–Overall Scale; SOC, standard of care.

**FIG. 4.**
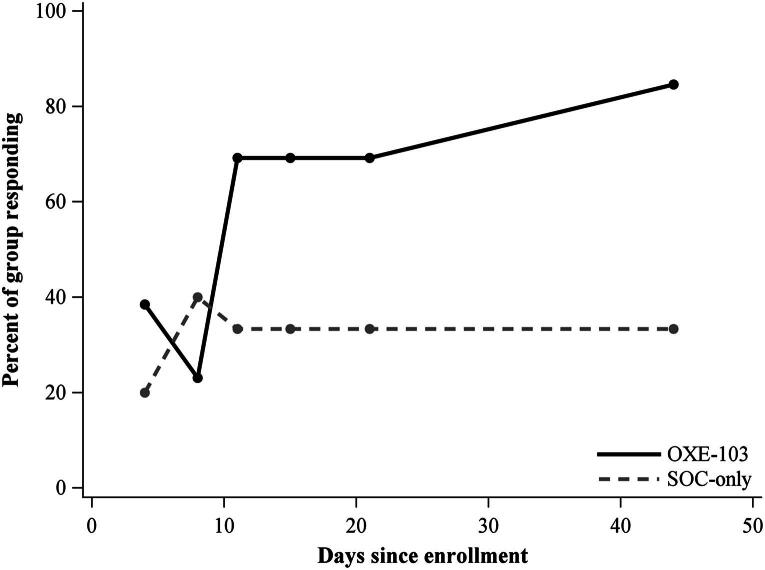
Participants with at least a 20% improvement in PCSS. PCSS, Post-Concussion Symptom Scale.

**FIG. 5.**
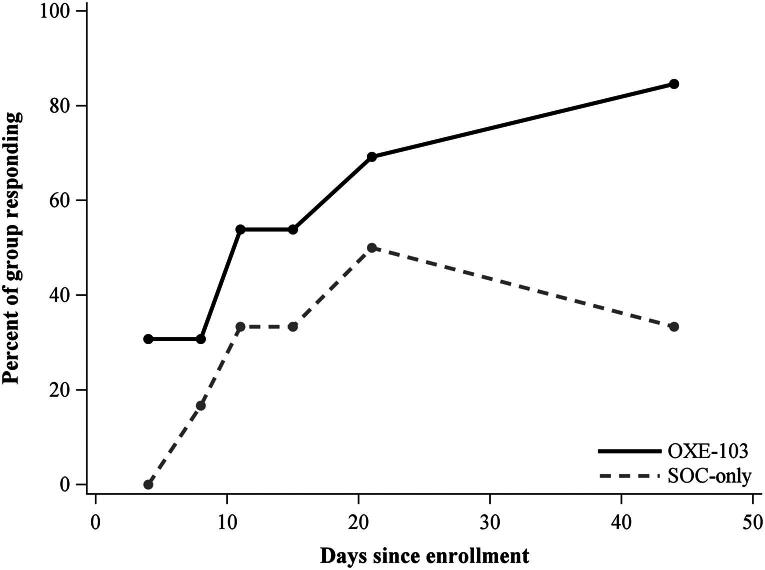
Participants with at least a 20% improvement in QOLIBRI. QOLIBRI, Quality of Life after Brain Injury–Overall Scale.

The PCSS responder rate at day 44 was 85% (95% CI: 53, 98) in the OXE-103 arm versus 33% (95% CI: 4, 78) in SOC-only arm. The QOLIBRI-OS responder rate at day 44 was also 85% (95% CI: 53, 98) in OXE-103 arm versus 33% (95% CI: 4, 78) in SOC-only arm (Figs. 4 and 5).

**FIG. 6.**
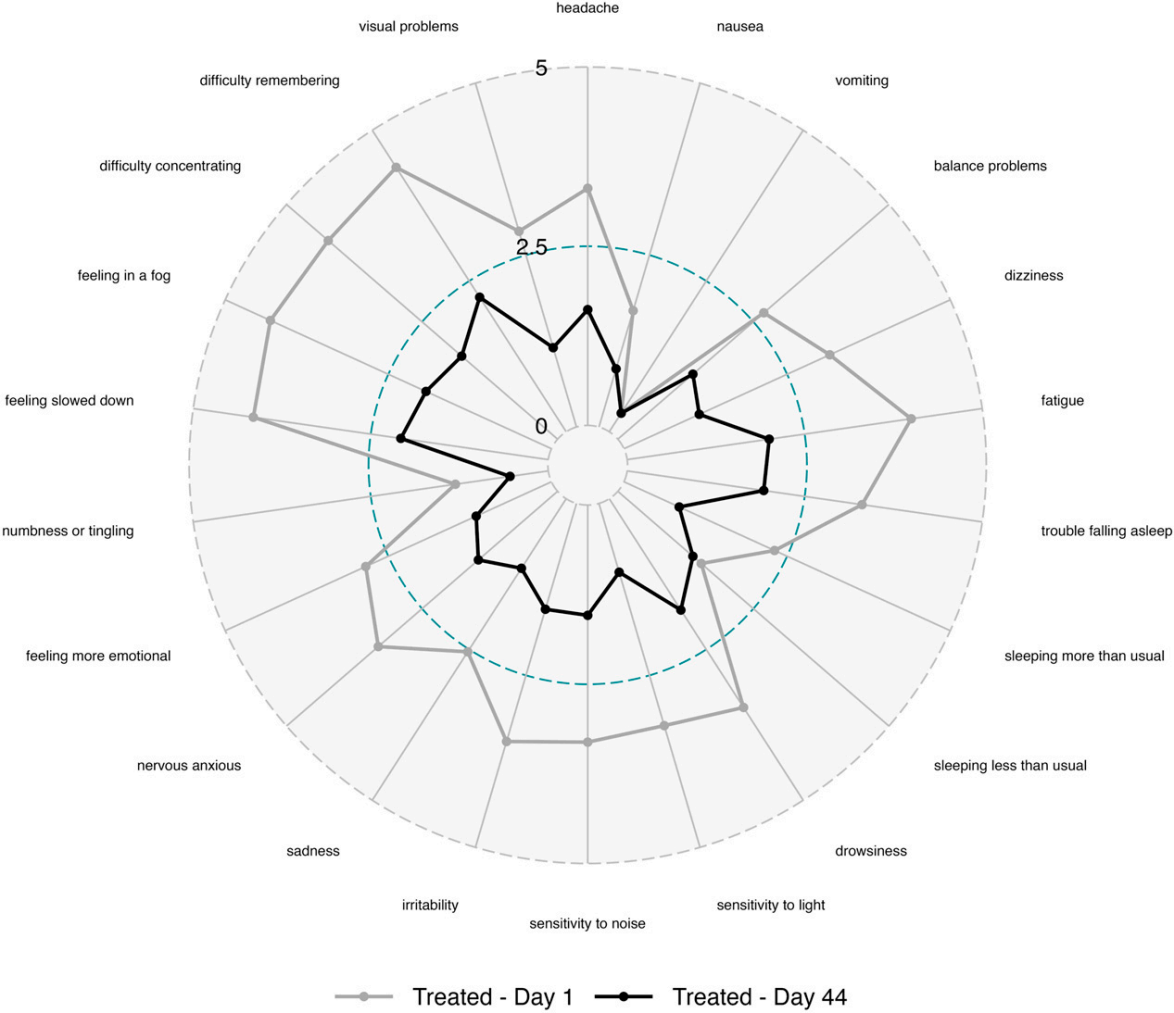
Change in individual symptom severity over time in OXE-103 group.

**FIG. 7.**
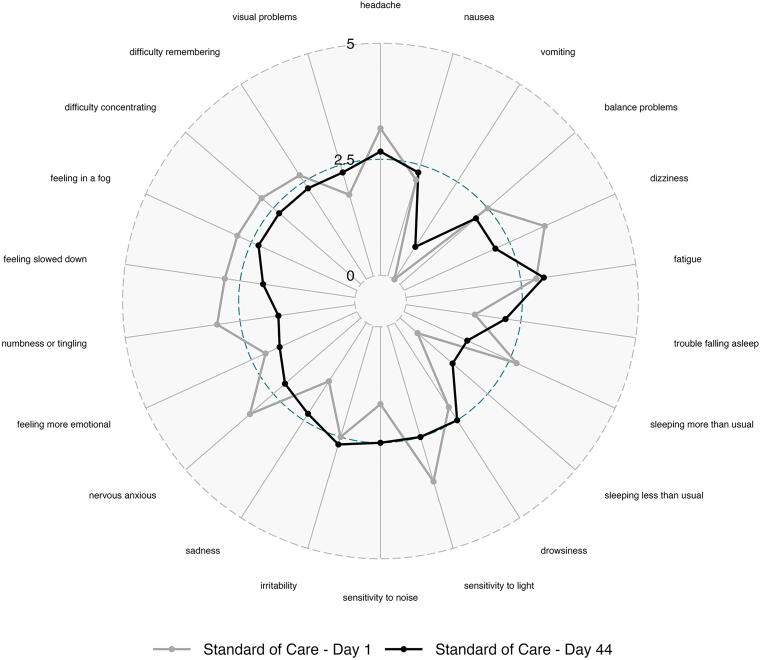
Change in individual symptom severity over time in SOC-only group. SOC, standard of care.

Severity ratings for each symptom on the PCSS were plotted to assess treatment effect across the spectrum of cognitive, emotional, and physical symptoms in the PCSS tool. Subjects treated with OXE-103 demonstrated improvement across the full spectrum of PCSS symptoms, except for sleeping less than usual and vomiting (Figs. 6 and 7).

The most commonly reported adverse events in the treatment group included injection related (bruising 53% and injection site pain 33%) and gastrointestinal related (increased appetite 53% and stomach gurgling 47%). This adverse event profile was consistent with the 300-patient safety database from previous studies of OXE-103 for other indications.^6^ There were no serious adverse events. There was no significant weight gain.

## Discussion

Here, we present a first-in-class clinical trial testing exogenous ghrelin therapy (OXE-103) as a treatment for concussions. Ghrelin has multiple effects on the central nervous system (CNS) beyond orexigenesis. Binding to GHSR-1a stabilizes the post-concussive neurometabolic aberrancies by increasing glucose availability, increasing mitochondrial-generated ATP, and decreasing ROS by UCP-2 pathway. Furthermore, ghrelin directly increases axonal potentiation and synaptic number, thereby increasing axonal connectivity.^3^ Ghrelin has also been shown to accelerate synapse formation and mediate neural plasticity after nervous system injury.^19^ Recent nonclinical studies of ghrelin as a neuroprotective intervention in several CNS models report increased regulated mitochondrial respiration and synaptic density, and reduced ROS, inflammation, and tau protein phosphorylation.^20–22^ Ghrelin has also been shown to decrease disruption of the blood–brain barrier in TBI, leading to a decrease in vascular permeability linked to decreased aquaporin-4 and S100B.^23^ Taken together, ghrelin has been shown to have effects on neurological energy homeostasis and neurogenesis. These effects are specifically relevant to limiting post-concussive metabolic impairment and its consequential neurotoxicity. However, what effect the hormone may have on post-concussive symptoms was unknown. Treatment with OXE-103 seems to be associated with a greater reduction in post-concussive symptom burden.

The primary outcomes of this study were improvement in symptoms and quality of life. The OXE-103 group shows a demonstrable decrease in symptoms reported on PCSS over the course of the study. This decrease is evident not only after the 2-week treatment period, but continues through the end of the study, 3 weeks after stopping OXE-103. Whereas the SOC group shows a relatively steady report of symptoms (Fig. 2). A similar trend is seen in quality of life reporting on QOLIBRI-OS. The OXE-103 group shows a notable increase in QOLIBRI-OS at the end of the 2-week treatment period with continued improvement during the 3-week follow-up period. However, the SOC group shows a generally unchanged QOLIBRI-OS (Fig. 3). We feel that both of these trends represent a possible therapeutic effect from OXE-103.

The research team chose to use 20% as the benchmark for clinically meaningful improvement on both the PCSS and QOLIBRI-OS. Over the first 4–8 days, the number of subjects who reported clinically meaningful improvements on PCSS was similar in both groups. However, by day 11, PCSS scores for the groups began to diverge, while QOLIBRI-OS scores maintained a moderate separation from days 11 to 21, then diverged at day 21. The SOC group had a consistent, clinically meaningful symptom improvement on PCSS in 33% of patients from day 10 through the end of the study period. In contrast, the OXE-103 cohort showed clinically meaningful symptom improvement in 69% of patients by day 10 and up to 85% of patients by the end of the study period (Fig. 4). There was a steady increase in clinically meaningful response by the OXE-103 group on QOLIBRI-OS. At the end of the study, 85% of the OXE-103 group showed a clinically meaningful response. This is in contrast to the SOC group, which showed only 33% of subjects with a clinically meaningful response at days 11, 15, and 44. There was an increase to 50% at day 21 before dipping back down to 33% at the conclusion of the study (Fig. 5). We feel that these trends also show a possible therapeutic response to OXE-103 that is clinically meaningful.

We also found that the trend of improvement on both PCSS and QOLIBRI-OS was sustained through day 44, both when looking at overall scores as well as the number of subjects with a clinically meaningful response. This is despite the cessation of OXE-103 administration at day 14. This may be due to sustained centrally mediated changes produced by OXE-103.

It’s unclear why the trends of clinically meaningful improvement on PCSS scores do not separate significantly until day 11. It is possible that the neurometabolic effects of OXE-103 may not result in an immediate symptom change. As mitochondrial generation and hippocampal volume increase, the biological effect may begin to diverge from the standard treatment cohort. Hippocampal growth and an increase in dendritic connections occurred after 5 days in one study.^5^ This may be similar to the clinical onset of some centrally acting psychiatric medications (e.g., monoamine oxidase inhibitors and selective serotonin reuptake inhibitors) occurring after 2 weeks.^24^

The particular symptoms and severity of each reported symptom varied significantly between subjects, which is a known phenomenon in concussed patients. However, the data suggest that treatment with OXE-103 affected all symptoms rather than a specific symptom or subset of symptoms. This is important as it suggests that OXE-103 is treating the underlying neurometabolic mechanism of injury rather than a particular symptom or system.

Our study was consistent with previous studies showing that the most common side effects are related to the injection site reactions and gastrointestinal symptoms, such as increased appetite and stomach gurgling. This would be consistent with the known effects of ghrelin, causing hunger and vagus nerve stimulation, which could lead to increased gastrointestinal peristalsis. There was no significant weight gain, likely due to the relatively short duration of treatment.

### Limitations

We recognize the limitations of this study, including a small sample size and the lack of randomization. While there was no randomization, the two cohorts still shared many similarities. The age of participants was similar in both groups and ranged from 30 to 59 years old. Both groups also had a similar length of days from injury, with medians of 25–26 days. The OXE-103 group had a higher PCSS at baseline; however, the SOC group had a higher QOLIBRI-OS at baseline.

The conversion to an open-label design increases the risk of placebo effect. A larger, randomized, placebo-controlled study would be required to adequately address this potential bias. However, we feel that the results of this study at least provide a signal of potential benefit, thereby justifying a larger study.

The potential for spontaneous recovery is another possible limitation for this study. In two studies, a higher initial PCSS score was correlated with prolonged symptomatology.^11,12^ Requiring a PCSS of at least 20, along with other inclusion and exclusion criteria, allowed us to enroll participants who are less likely to spontaneously improve. Our participants had PCSS scores much higher than 20, therefore, we feel confident that our cohort is one of persistence. It could also be expected that spontaneous recovery could occur equally in both groups. Despite this, greater improvement was observed in participants treated with OXE-103 compared to the SOC-only group.

We recognize that self-reported symptom assessments could be considered as a limitation. However, we firmly believe that self-reported outcomes such as symptom burden and quality of life are important to measure the impact of treatment, given the lack of definitive objective measures. These self-reported metrics are also consistent across the range of clinical studies in concussion, neuropsychiatry, and pain. Blood biomarkers, neuroimaging (e.g., DTI, MRS, fMRI), neurocognitive testing, and vestibular testing may augment future studies as a more objective metric, although the reduction of patient symptoms and improvement in quality of life is still the most clinically relevant outcome. Future studies will need to be more robust with additional dosing and dosing duration arms.

Additionally, there were several interesting lessons and insights gained in conducting this trial. Unlike many previous concussion studies, we were able to target a cohort that was not solely a sports-related concussion but rather from a variety of injury mechanisms. We feel this population more readily represents the general public, where the majority of concussions occur. The participants were largely women, which is also an underrepresented population in previous concussion research.

Some eligible subjects expressed a lack of interest in enrolling in a randomized, placebo-controlled design. While common for placebo-controlled studies, this raises some interesting questions about future studies and their design. This may necessitate a study design that incorporates a crossover design where both groups receive treatment and placebo, but at differing times. Other considerations would be converting the placebo group to treatment after a predefined time.

## Conclusions

Treatment with OXE-103 showed a possible treatment effect that was observed across a spectrum of endpoints. Primarily, subjects treated with OXE-103 showed improved PCSS and QOLIBRI-OS scores compared to those receiving only standard therapy. OXE-103 also appears to provide relief for a spectrum of symptoms rather than symptom-directed therapies. While the subjects receiving OXE-103 seemed to receive a greater benefit than the SOC-only group, sample size is small, and randomization was not used. Nevertheless, this preliminary data is compelling as a potential first-in-class treatment for post-concussion symptomatology. Larger studies are needed.

## Transparency, Rigor, and Reproducibility Summary

The study design and analysis plan were preregistered on ClinicalTrials.gov (NCT04558346). The analysis plan was not formally preregistered, but the team member with primary responsibility for the analysis (e.g., biostatistician, lead author) certifies that the analysis plan was prespecified. Prespecified sample size was 20 per group; however, actual sample size was 13 in the treatment group and 6 in the SOC group. A total of 25 subjects enrolled with 19 completing all study procedures; 13 received OXE-103 treatment and 6 SOC-only (Table 1). One subject withdrew after consenting but prior to completing any study activities. One subject withdrew after completing baseline activities but prior to receiving any study treatment. Two subjects consented and received some treatment but withdrew before study completion. One SOC-only participant was lost to follow-up. One was randomized to placebo early on and completed the study but is not included in the analysis. Participants were not randomized and were initially offered enrollment in the treatment arm and subsequently the SOC arm. Investigators were not blinded due to the design of the study and the lack of placebo/sham. All materials required to perform the outcome assessments are widely available on the internet and considered standard in the field. Material used for therapeutic intervention (OXE-103) came from the supply of Oxeia Biopharmaceuticals. Statistical analysis was performed by Suzanne Hunt, who is a trained statistician. Methods for multiple comparisons were not necessary. The findings have not yet been replicated or externally validated. De-identified data from this study are not available in a public archive. De-identified data from this study will be made available (as allowable according to institutional IRB standards) by emailing the corresponding author. The authors agree or have agreed to publish the article using the Mary Ann Liebert Inc. “Open Access” option under an appropriate license.

## Abbreviations Used

ATP: adenosine triphosphate
CNS: central nervous system
GHSR: growth hormone secretagogue receptor type-1
IQR: Interquartile range
IRB: institutional review board
PCSS: Post-concussion symptom scale
QOLIBRI-OS: Quality of Life After Brain Injury – Overall Scale
ROS: reactive oxygen species
SOC: Standard of Care
TBI: traumatic brain injury
UCP-2: mitochondrial uncoupling protein 2
