# Supplementary material for: The Injection of Ghrelin (OXE-103) Improves Subacute Concussion Symptom Burden and Quality of Life
Source: Neurotrauma Rep. 2025 May 16;6(1):402–12. doi: 10.1089/neur.2025.0038 (PMC12281116; doi:10.1089/neur.2025.0038)

Supplemental Data

Individual PCSS scores of participants at days 1, 15, and 44

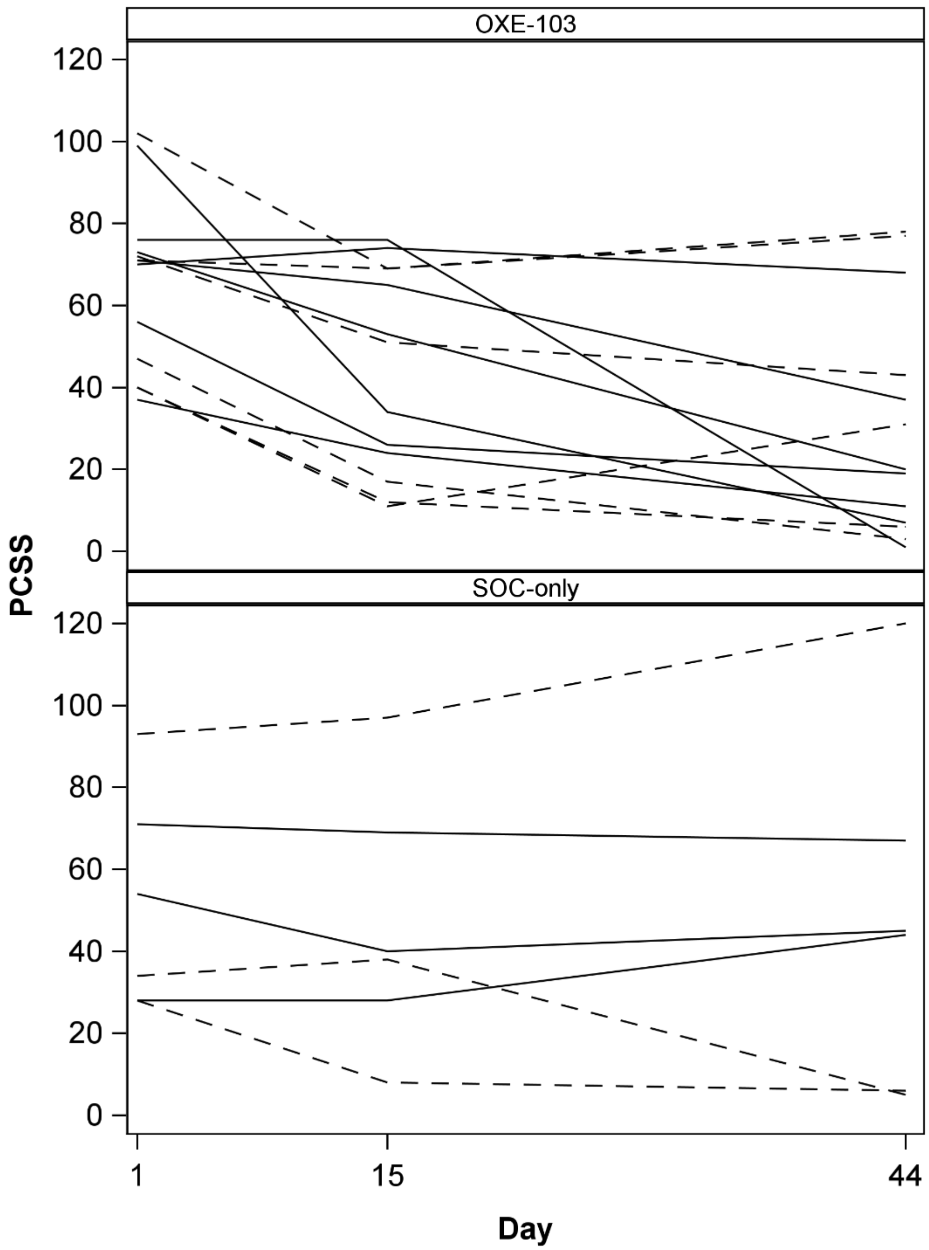

Individual QOLIBRI-OS scores of participants at days 1, 15, and 44

*2 participants in the OXE-103 group had the same scores of 0 (day 1), 0 (day 15), and 50 (day 44) thus they share a line

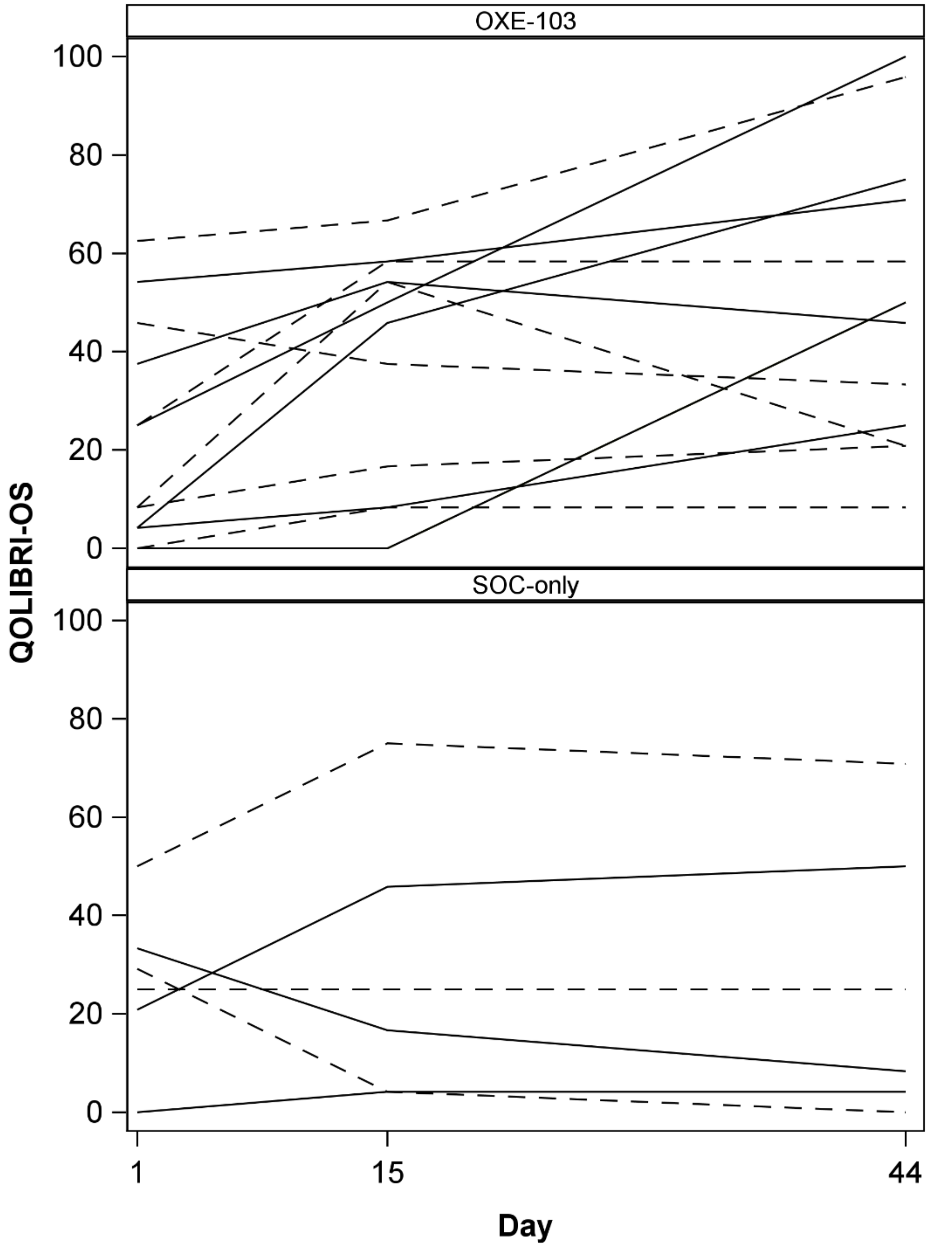

Supplement: Supplementary Data [file neur.2025.0038_supplementarydata.pdf]
